# The Impact of High-Intensity Interval Training on Brain Derived Neurotrophic Factor in Brain: A Mini-Review

**DOI:** 10.3389/fnins.2018.00839

**Published:** 2018-11-14

**Authors:** Alberto Jiménez-Maldonado, Iván Rentería, Patricia C. García-Suárez, José Moncada-Jiménez, Luiz Fernando Freire-Royes

**Affiliations:** ^1^Facultad de Deportes, Universidad Autónoma de Baja California, Ensenada, Mexico; ^2^Human Movement Sciences Research Center, University of Costa Rica, San José, Costa Rica; ^3^Laboratório de Bioquímica do Exercício, Universidade Federal de Santa Maria, Santa Maria, Brazil

**Keywords:** brain-derived neurotrophic factor, high-intensity interval training, brain, health, physical exercise

## Abstract

The brain-derived neurotrophic factor (BDNF) is a protein mainly synthetized in the neurons. Early evidence showed that BDNF participates in cognitive processes as measured at the hippocampus. This neurotrophin is as a reliable marker of brain function; moreover, recent studies have demonstrated that BDNF participates in physiological processes such as glucose homeostasis and lipid metabolism. The BDNF has been also studied using the exercise paradigm to determine its response to different exercise modalities; therefore, BDNF is considered a new member of the exercise-related molecules. The high-intensity interval training (HIIT) is an exercise protocol characterized by low work volume performed at a high intensity [i.e., ≥80% of maximal heart rate (HRmax)]. Recent evidence supports the contention that HIIT elicits higher fat oxidation in skeletal muscle than other forms of exercise. Similarly, HIIT is a good stimulus to increase maximal oxygen uptake (VO_2_max). Few studies have investigated the impact of HIIT on the BDNF response. The present work summarizes the effects of acute and long-term HIIT on BDNF.

## Introduction

Physical exercise (PE) is considered a subcategory of the physical activity domain (Caspersen et al., 1985; Physical Activity Guidelines Advisory Committee, 2008). As opposed to physical activity, PE is characterized for being planned, structured, and repetitive, with the inherent goal of improving one or more components of the physical fitness, physical performance, or health (Caspersen et al., 1985;Physical Activity Guidelines Advisory Committee, 2008). The PE prescription is usually reported as exercise mode, intensity, frequency, and duration of the activity.

Several training modalities have been developed over the years with the aim of improving cardiorespiratory fitness, musculoskeletal function, and metabolic activity. Among these, aerobic, endurance, and/or resistance (i.e., strength exercise) are the most common exercise training modalities (Kang and Ratamess, 2014). The aerobic exercise (AE) is also known as moderate-intensity continuous training (MICT), and it is usually performed over long periods of time (e.g., ≥30-min to moderate intensity, performing exercises such as walking, cycling, jogging, and swimming) (Garber et al., 2011). Although the benefits of MICT on health related parameters in humans are well studied (Aldred et al., 1995; Poehlman et al., 2000; Mador et al., 2004; Frøsig et al., 2007; Camargo et al., 2008; Bell et al., 2010; Fisher et al., 2015; Daabis et al., 2017); currently, the world population considers that lack of time as the main barrier to practice AE regularly (Weston et al., 2014; Fisher et al., 2015).

Scientists and exercise professionals have focused on studying the impact of short exercise bouts on human physiology with the aim of optimizing time use (Gibala et al., 2006); for instance, high-intensity interval training (HIIT). The HIIT refers to exercise characterized by relatively short bursts of vigorous activity, interspersed by rest or low-intensity recovery exercise. In general, HIIT is performed on a training session lasting ≤30-min, including warm-up and cool down stages (Gibala and Jones, 2013; Gillen and Gibala, 2013; Weston et al., 2014). The high-intensity bouts should be performed at near maximal effort, reaching intensities between 80 and 100% of the maximal heart rate (HRmax) (Gibala et al., 2014; Saanijoki et al., 2018). The exertion is performed no longer than 60-s (Gillen and Gibala, 2013), and the recovery periods (low-intensity exercise or rest) can be up to 4-min (Burgomaster et al., 2005, 2006; Gibala and McGee, 2008). Besides Gibala’s protocols, others have reported different high- and low-exercise bout durations (Saucedo Marquez et al., 2015; Lira et al., 2017; Stöggl and Björklund, 2017); however, the training session has been kept within ≤30-min.

In addition, HIIT can be performed on cyclical exercises such as bicycling (Saucedo Marquez et al., 2015), running (Lira et al., 2017), swimming (Courtright et al., 2016), and whole-body exercise (Machado et al., 2017; Schleppenbach et al., 2017). Several physiological adaptations of HIIT have been reported to improve physical performance in humans (Burgomaster et al., 2005, 2006; Gibala et al., 2006; Talanian et al., 2007; Connolly et al., 2017). The effects of HIIT on brain function have been also reported; however, there available evidence is scarce (Afzalpour et al., 2015; Lucas et al., 2015; Coetsee and Terblanche, 2017; Santos-Concejero et al., 2017; So et al., 2017; Freitas et al., 2018; Robinson et al., 2018). Indeed, the current evidence showed a positive impact of HIIT in brain, specifically in neurotrophin expression and function. In this context, the aim of this work is to briefly describe the current knowledge regarding the acute and long-term effects of HIIT on brain-derived neurotrophic factor (BDNF) in brain. It is known that BDNF is a protein that plays a key role to maintain or improve several brain functions (Vaynman et al., 2003, 2004; Duman and Monteggia, 2006; Duman and Li, 2012; Fernandes et al., 2017).

## High-Intensity Interval Training (HIIT): an Efficient Tool to Improve Physical Performance, Metabolism, and Brain Function

As described, HIIT refers to exercise characterized by relatively short bursts of vigorous activity, interspersed by rest or low-intensity recovery exercise (Gibala and Jones, 2013; Gillen and Gibala, 2013; Weston et al., 2014). Previous scientific reports have indicated that HIIT is perceived as an exercise modality eliciting higher exhaustion compared to MICT (Saanijoki et al., 2015, 2018). However, HIIT is considered more enjoyable than MICT (Heisz et al., 2016); in agreement with this, HIIT has been proposed as an excellent strategy aimed to increase adherence to exercise programs in sedentary people (Heisz et al., 2016). In this section, the impact of HIIT on the human physiology (physical performance, metabolism, and brain function) will be briefly described. In sport, scientific reports indicate that HIIT was popularized by the runner Emil Zatopek around 1950 [see Billat (2001) review on the historical analysis of HIIT]; in fact, several coaches think that HIIT played a key role on Zatopek’s successful sport career. Similarly, recent evidence indicates that HIIT improves physical performance (e.g., speed, agility) in team sport athletes such as soccer and basketball (Iaia et al., 2015; Sanchez-Sanchez et al., 2018).

Regarding to metabolic dysfunctions in glucose and lipids induced by sedentary lifestyle and hypercaloric diets, several evidence showed that HIIT is an efficient stimulus to improve lipid and glucose metabolism. Concretely, Talanian et al. (2007), reported that seven sessions of HIIT increased fat oxidation in skeletal muscle in recreationally active women. Similarly, others demonstrated that HIIT interventions enhance insulin sensitivity, glucose control, and cardiorespiratory fitness in sedentary women (Connolly et al., 2016, 2017). In addition to research on women, others reported that HIIT increases muscle oxidative capacity in recreationally active men (Burgomaster et al., 2005, 2006; Gibala et al., 2006).

The central nervous system (CNS) response to HIIT has been reported in spinal cord (Astorino and Thum, 2018) and brain studies (Coetsee and Terblanche, 2017; Santos-Concejero et al., 2017; Robinson et al., 2018). For instance, a 16-week HIIT program elicited higher oxygen utilization and cerebral oxygenation than MICT in older people (Coetsee and Terblanche, 2017); similar results were found in younger adults (Robinson et al., 2018). In these studies, the BDNF’s response was dependent of the exercise intensity. However, the molecular mechanisms explaining these brain adaptations to HIIT are yet to be elucidated.

## Brain-Derived Neurotrophic Factor (BDNF): a Protein Sensitive to Exercise in Brain

### BDNF Function in Brain and Periphery

The BDNF is a protein member of the neurotrophin family, and it is found in the nervous system and peripheral organs such as skeletal muscle (Funakoshi et al., 1993; Conner et al., 1997; Matthews et al., 2009). In the CNS, the neurons are the principal source of BDNF (Mowla et al., 2001), and evidence suggests that BDNF plays a key role in memory and learning processes (Erickson et al., 2011). Moreover, molecular evidence indicates that this neurotrophin through a tyrosine kinase b receptor (TrkB) increases long-term potentiation, neurogenesis, axonal growth, and synaptogenesis (Tyler and Pozzo-Miller, 2001; Vaynman et al., 2003, 2004; Fernandes et al., 2017). Besides the local effect of BDNF in the brain, some authors suggest that the brain is the major source of circulating BDNF at rest and during exercise (Rasmussen et al., 2009; Seifert et al., 2010). In the periphery, studies performed in rodent and human tissues have revealed that BDNF regulates other physiological pathways such as glucose metabolism (Hanyu et al., 2003; Jiménez-Maldonado et al., 2014), and fat oxidation (Matthews et al., 2009).

### Molecular Mechanism Induced by Physical Exercise Increasing Brain BDNF

Several stimuli can increase BDNF’s expression and function. In rodents, the kainic acid exposure increased hippocampal BDNF (protein) levels (Rudge et al., 1998), resulting from an enhancement in glutamatergic signaling. Other evidence suggests that intermittent hypoxia increases BDNF levels in neurons of the primary motor cortex (Satriotomo et al., 2016). In addition to these findings, it is widely known that PE is an effective stimulus to increase BDNF synthesis in the brain (Oliff et al., 1998; Vaynman et al., 2003, 2004; Erickson et al., 2011), and the periphery (Dinoff et al., 2016, 2017).

**FIGURE 1:**
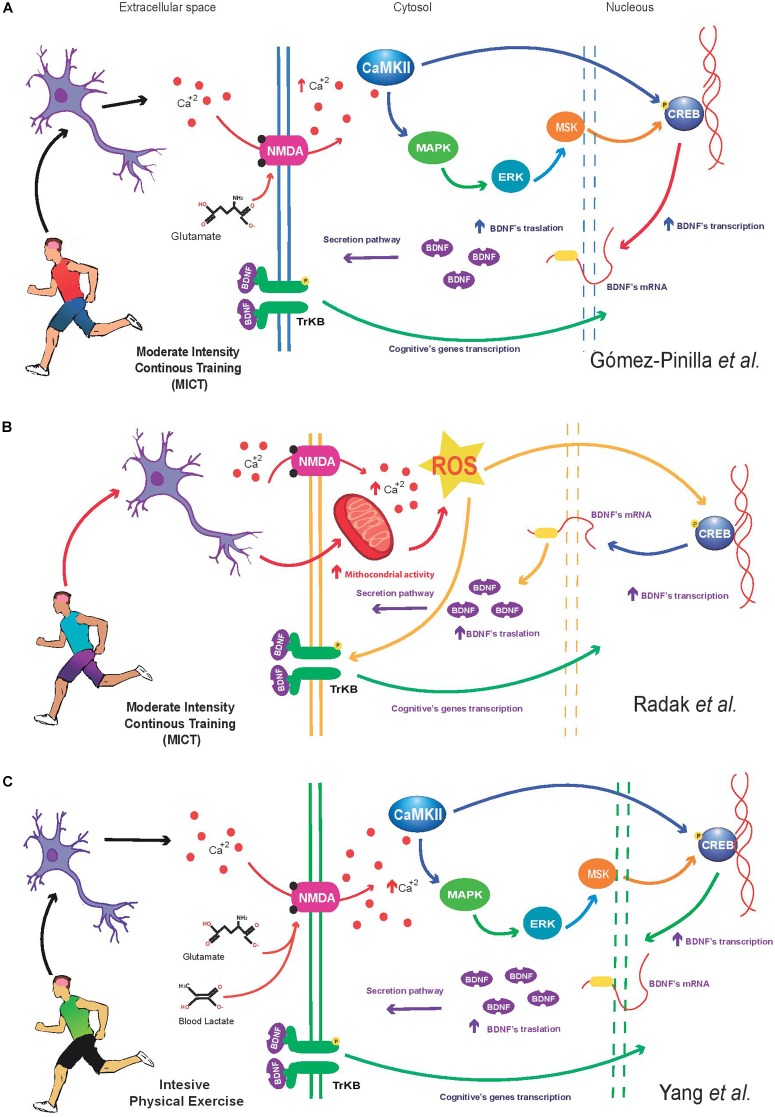
**(A)** Moderate-intensity continuous training (MICT) increases intracellular calcium (Ca^2+^) levels in neurons through the NMDA receptor. Intracellular Ca^2+^ increases the activity of calmodulin dependent kinase II (CaMKII), triggering the activation of the MAPK/ERK/MSK cascade signaling, resulting in the increase of the expression and phosphorylation of cAMP response element-binding protein (CREB). Finally, CREB enhance the *Bdnf* transcription. This molecular mechanism described above result in a higher BDNF protein, the neurotrophin is released by the neuron to induce transcription of cognitive genes. The present model is based on Gomez-Pinilla’s studies (Fernandes et al., 2017). **(B)** MICT enhances the mitochondrial activity in neurons. Higher mitochondrial activity increases reactive oxygen species (ROS) from complexes I and III. The change in ROS levels modify and regulate a wide of signaling process including the CREB-BDNF signaling pathway. Once activated, BDNF regulates a positive feedback mechanism to induce the cognitive genes transcription. Additionally, the aerobic exercise increases the calcium concentration in neurons; this ion through the calpain and xanthine oxidase increases the ROS that consequently increase the CREB’s activation and *Bdnf* expression. **(C)** Exercise performed at high intensity (≥80% HRmax) activates several metabolic pathways in muscle (including glycolysis), this condition generates a higher systemic blood lactate concentration reaching the brain, this metabolite can be oxidized by astrocytes or neurons to produce glucose (Dienel and Hertz, 2001). In addition, experimental evidence indicates that lactate increase NMDA activity and intracellular Ca^2+^ levels in neurons. Indeed, it is possible that the lactate in neurons enhance the CaMKII activity and the MAPK/ERK/MSK signaling to induce the CREB’s activation and *Bdnf* expression. Finally, the BDNF activate a positive loop to induce the expression of cognitive genes (Yang J. et al., 2014).

Regarding to the impact of the PE on increasing BDNF in brain, different molecular mechanisms have been proposed to explain how PE (mainly MICT) enhances BDNF synthesis in neurons. The Gomez-Pinilla’s group suggests that PE increases the intracellular Ca^2+^ levels in neuronal cells (Fernandes et al., 2017). This ion activates CaMKII indirectly; and once active, this kinase increases the MAP-K pathway to phosphorylate CRE-binding protein and activate the CREB transcription, and consequently *Bdnf* transcription (Vaynman et al., 2004; Fernandes et al., 2017; Figure 1A). Another model suggests that PE induces BDNF synthesis in the brain by enhancing the activity of reactive oxygen species (ROS) (Radak et al., 2016). Based on Radak et al’s. proposal, PE increases the mitochondrial activity in neurons; and it is known that higher mitochondrial activity produce excessive ROS. Thus, ROS enhance the activity of CRE-binding protein, to activate the CREB and *Bdnf* transcription (Radak et al., 2016; Figure 1B). Additionally, the Radak’s model indicates that the exercise increases the Ca^2+^ in neurons; this ion through the calpain and xanthine oxidase induces higher ROS production in brain as well (Takuma et al., 1999; Kahlert et al., 2005). In addition to the previous mechanism, it has been suggested that systemic molecules such as the lactate synthesized during intensive PE (≥80% HRmax) can activate the BDNF production (Bergersen, 2015). However, this molecular mechanism of BDNF production is still poorly understood. Experimental evidence has shown higher NMDA receptor activity in the presence of lactate; furthermore, high lactate concentrations are associated to increased neuronal Ca^2+^ levels (Yang J. et al., 2014) and higher *Bdnf* transcription (Yang J.L. et al., 2014). It is likely that lactate synthesized during PE reach the neurons and increase the NMDA receptor activity to increase the Ca^2+^ concentration in neurons, and Ca^2+^ activates CaMKII, and consequently, the kinase phosphorylates activating the MAPK/ERK signaling pathway to enhance *Bdnf* transcription (Figure 1C).

## The Effects of Hiit on Bdnf

### Animal Models

As previously described, HIIT is characterized by exercise bouts of high-intensity and low volume. Regarding exercise intensity, evidence in healthy rodents has shown that brain BDNF synthesis was higher in animals performing a high-intensity training compared to those animals performing a low-intensity training and sedentary rodents (de Almeida et al., 2013). However, the authors used a continuous training protocol; the training time was similar in both models (low- and high-intensity training, 30-min/session), which means that the HIIT characteristics were unattained.

There is evidence regarding the long-term effects of HIIT on BDNF synthesis in rodents (Afzalpour et al., 2015; So et al., 2017; Freitas et al., 2018). Thirty HIIT sessions significantly increased BDNF levels (protein) in the brain compared with continuous training protocol and a control group (Afzalpour et al., 2015). The authors discussed that HIIT increased hydrogen peroxide (H_2_O_2_) and Tumor Necrosis Factor Alpha (TNF-α) concentration in brain; and these molecules could activate the BDNF synthesis (Wang et al., 2006; Bałkowiec-Iskra et al., 2011) or CREB (Pugazhenthi et al., 2003), a transcription factor positively regulating BDNF synthesis. However, although the previous paper found a positive effect of HIIT on BDNF, the authors did not report a specific anatomical region sensitive to elevations on the neurotrophin following HIIT. Consequently, others evaluated with more detail the impact of HIIT on BDNF in the hippocampus (Freitas et al., 2018). In the study by Freitas et al. (2018), 36 sessions of HIIT elevated BDNF levels in the hippocampal region of healthy rats. However, the molecular mechanism responsible for increasing BDNF synthesis was not demonstrated in the study. In agreement with their results, the authors suggested that 36 HIIT sessions increased BDNF levels and attenuated hippocampal oxidative damage (Freitas et al., 2018).

### Human Models

There are reports on the effect of a single HIIT session on BDNF (Saucedo Marquez et al., 2015; Cabral-Santos et al., 2016; Slusher et al., 2018). For instance, a single session of supramaximal HIIT elevated serum BDNF levels (Slusher et al., 2018), suggesting increases in BDNF secretion of the platelets unrelated to brain secretion (Slusher et al., 2018). Saucedo Marquez et al. (2015), found that HIIT was a more powerful stimulus to elevate systemic (serum) BDNF compared to MICT. The exercise modality employed in their study (cycle-ergometer) did not induce muscle damage (Saucedo Marquez et al., 2015). Therefore, the higher BDNF levels were not caused by platelet activation to increase the BDNF secretion (Saucedo Marquez et al., 2015), suggesting that PE itself is enough stimuli that lead to higher circulating BDNF levels. Thus, the higher serum BDNF levels following HIIT resulted from a greater synthesis of BDNF in the brain. The authors discussed that a single bout of HIIT induced higher brain H_2_O_2_ and TNF-α levels. These molecules activate the signaling of peroxisome proliferator-activated receptor-γ coactivator (PGC-1α) to enhance neuron BDNF synthesis (Saucedo Marquez et al., 2015). Similarly, a single session of HIIT significantly increased peripheral plasmatic BDNF levels immediately following the exercise (Cabral-Santos et al., 2016). However, after 60-min that the HIIIT session ended, BDNF concentrations returned to baseline levels (Cabral-Santos et al., 2016). Regarding that plasmatic BDNF levels reflect the BDNF secretion from the brain (Lommatzsch et al., 2005); the Cabral-Santos data reflect the HIIT impact on BDNF in brain. The authors suggested that brain hypoxia induced by HIIT was the main factor explaining their results (Cabral-Santos et al., 2016).

Finally, the long-term effects of HIIT on systemic (serum) BDNF levels have been also reported (Murawska-Cialowicz et al., 2015). In the study, participants performed whole-body exercises for 3 months, and the protocol was effective at increasing serum BDNF concentrations. However, the BDNF source was not elucidated.

## Perspectives and Concluding Remarks

**FIGURE 2:**
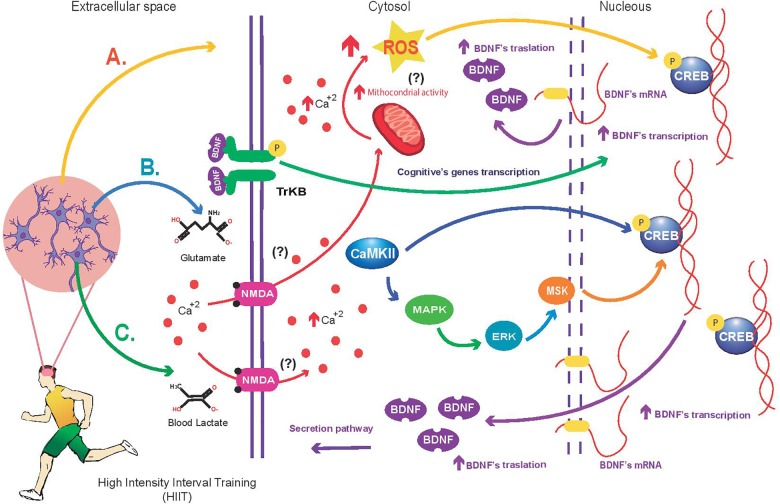
Mechanism proposed about the High Intensity Interval Training (HIIT) impact on BDNF synthesis in brain. **(A)** HIIT increases mitochondrial activity (not reported) and ROS concentration in neurons (Afzalpour et al., 2015) compared with MICT. ROS induce higher *Creb-Bdnf* transcription and signaling than MICT (not reported). **(B)** HIIT causes greater Ca^2+^ concentration in neurons than MICT (not reported); this condition enhances the CaMKII activity and MAPK/ERK/MSK signaling to activate the *Creb-Bdnf* transcription and neuronal plasticity. Additionally, the intracellular calcium can increase the ROS generation in neurons. Once synthetized, ROS can activate *Creb-Bdnf* transcription. Currently, there is not experimental evidence to indicate that HIIT triggers more this mechanism than MICT. **(C)** HIIT elevate systemic blood lactate concentration, and consequently enhance the NMDA receptor activity to increase intracellular Ca^2+^ concentration in neurons (not reported). The ion activates the CaMKII activity and MAPK/ERK/MSK signaling to induce the *Creb-Bdnf* transcription and neuronal plasticity (not reported). (?): not reported.

Studies performed in rodents (Tsuchida et al., 2001; Hanyu et al., 2003; Yamanaka et al., 2006; Jiménez-Maldonado et al., 2014) and humans (Bulloì et al., 2007; Krabbe et al., 2007; Li et al., 2016) have demonstrated that BDNF participates in glucose and lipid metabolism (Matthews et al., 2009). Therefore, this molecule also is known as metabotrophin (Gomez-Pinilla et al., 2008). Several health conditions such as type 2 diabetes, obesity, metabolic syndrome, and cardiovascular diseases are mainly caused by dysfunctional metabolic mechanisms and sedentary lifestyle. Therefore, it is important to identify efficient stimuli to increase the BDNF function in population with high risk to suffer metabolic diseases or in people who are suffering metabolic diseases. Thus, HIIT seems be a good stimulus to enhance the BDNF action. However, the impact of HIIT on BDNF and its effect on glucose and lipid metabolism is poorly studied. Further experimental studies are necessary to elucidate the impact of HIIT on BDNF and its effect on glucose and lipid metabolism in humans with metabolic or cardiovascular diseases. In addition, during modern-life diseases (Type II diabetes, obesity, and metabolic syndrome); the brain function is also affected (Cisternas et al., 2015; Agrawal et al., 2016). Therefore, studies are needed to assess the impact of HIIT interventions on BDNF synthesis and signaling pathways in brain under morbid conditions. The current work proposes a model about the impact of HIIT on BDNF expression in the brain (Figure 2). It will be reasonable to use previous HIIT protocols that reported a positive impact in peripheral BDNF when thinking about the design of HIIT protocols aimed at increasing BDNF synthesis and brain signaling. For example, sprint interval training (60-s run at 100% VO_2_max, interspersed with 60-s passive recovery) (Cabral-Santos et al., 2016). In addition, the peak power output (PPO-Watts-) can also be used to design a HIIT protocol (Saucedo Marquez et al., 2015); for instance, the protocol could consist in pedaling for 60-s at 90% of PPO, alternating with 60-s of active rest at 60 Watts (total duration of HIIT is 20-min) (Saucedo Marquez et al., 2015). Finally, a recent report performed in overweight subjects showed that a HIIT protocol designed using heart rate as the main variable to establish the workload intensities is not a good stimulus to increase the peripheral BDNF (Domínguez-Sanchéz et al., 2018). Further studies are needed to determine whether heart rate may be considered as a reliable physiological variable used to design a HIIT protocol aimed at increasing circulating BDNF in non-obese subjects.

## Author Contributions

AJ-M conceived the review focus, reviewed the literature, wrote the first draft, and finalized the manuscript. IR and PG-S reviewed the literature, wrote the first draft, and finalized the manuscript. JM-J and LF-R finalized the manuscript. All authors approved the final version of the manuscript.

## Conflict of Interest Statement

The authors declare that the research was conducted in the absence of any commercial or financial relationships that could be construed as a potential conflict of interest.
